# Obstacles and Solutions Driving the Development of a National Teleradiology Network

**DOI:** 10.3390/healthcare9121684

**Published:** 2021-12-06

**Authors:** Leonie Goelz, Holger Arndt, Jens Hausmann, Christian Madeja, Sven Mutze

**Affiliations:** 1 Department of Radiology and Neuroradiology, BG Klinikum Unfallkrankenhaus Berlin, Warener Straße 7, 12683 Berlin, Germany; holger.arndt@ukb.de (H.A.); jens.hausmann@ukb.de (J.H.); christian.madeja@ukb.de (C.M.); sven.mutze@ukb.de (S.M.); 2 Institute for Diagnostic Radiology and Neuroradiology, University Medicine Greifswald, Ferdinand-Sauerbruch-Straße, 17475 Greifswald, Germany

**Keywords:** teleradiology, national, network, obstacles, solutions

## Abstract

Background: Teleradiology has the potential to link medical experts and specialties despite geographical separation. In a project report about hospital-based teleradiology, the significance of technical and human factors during the implementation and growth of a teleradiology network are explored. Evaluation: The article identifies major obstacles during the implementation and growth of the teleradiology network of the Berlin Trauma Hospital (BG Unfallkrankenhaus Berlin) between 2004 and 2020 in semi-structured interviews with senior staff members. Quantitative analysis of examination numbers, patient numbers, and profits relates the efforts of the staff members to the monetary benefits and success of the network. Identification of qualitative and quantitative factors for success: Soft and hard facilitators and solutions driving the development of the national teleradiology network are identified. Obstacles were often solved by technical innovations, but the time span between required personal efforts, endurance, and flexibility of local and external team members. The article describes innovations driven by teleradiology and hints at the impact of teleradiology on modern medical care by relating the expansion of the teleradiology network to patient transfers and profits. Conclusion: In addition to technical improvements, interpersonal collaborations were key to the success of the teleradiology network of the Berlin Trauma Hospital and remained a unique feature and selling point of this teleradiology network.

## 1. Introduction

### 1.1. Teleradiology as Basic Necessity

The origins of teleradiology date back to the 1960s and 1970s, during which transmission of X-ray imaging was achieved using microwaves. Pioneers in this field aimed to cover a physical distance, which enabled reading and reporting from remote localization [1]. From early days on, radiologists and technicians have struggled to balance technological innovations in radiology and necessary processes to im- and export imaging.

The development of teleradiology is driven by the need to provide specialized radiological care in sparsely populated areas at any time of the day and to meet increasing economic expectations [2]. Rural depopulation is a growing issue in many areas worldwide, such as Europe, China, Japan, and the United States of America, and is a major driving force for technical improvements in teleradiology [3,4,5,6,7].

### 1.2. Milestones of Teleradiology Development

The milestones for the expansion of teleradiology were described in detail in 2007 [2]. In brief, the digitalization of radiological imaging and the ability to store and review images conveniently in picture archiving and communication systems (PACS) were key factors for efficient reporting from a distance [8,9]. Technical limitations repressing and slowing the rise of teleradiology were identified in display technique, processor speed, data transmission, and data storage infrastructure [10]. Gradual adaptation and stepwise integration of technological communication standards such as Digital Imaging and Communications in Medicine (DICOM) or Health Level 7 (HL7) and the need to preserve medical records and imaging at any cost account for variable depths of integration of teleradiological sites into radiology and hospital information systems (RIS and HIS) [11]. At certain points in time, inhomogeneous networks can therefore appear as patchwork solutions, which ultimately require heightened efforts to facilitate and accelerate the management of cases [12]. In fact, a 2012 European survey found that even though widely used and accepted, most teleradiology was conducted via noncommercial services [13].

Ongoing concerns of teleradiology operators are persistent technologic limitations [14], further distancing radiologists from patient care [15], the inclusion of economically underdeveloped countries with affordable systems [16], privacy and data security issues [17], and cross-border teleradiology [18,19].

Some modern technological approaches in teleradiology focus on facilitating communication between healthcare professionals and patients. The TK med system is a web-based program that upgrades existing teleradiology systems and allows an exchange between all parties involved in patient care [20]. Other developers are working on direct integration of intelligent deep learning applications directly into RIS/PACS architectures [21]. Current trends in teleradiology propagate the division of radiological and technical expertise. Tech companies can provide cloud-based architectures that store medical data and deliver imaging to their customers [22]. Future developments might show whether data protection requirements and national laws will support the expansion of such solutions.

### 1.3. Teleradiology Network of Berlin Trauma Hospital

As a city-state and the capital of Germany, Berlin is the German city with the highest population, with 3.66 million inhabitants. Berlin is surrounded by Brandenburg, a state with one of the lowest population densities in the country (approximately 85 inhabitants per square kilometer) [23].

Berlin Trauma Hospital (BG Klinikum Unfallkrankenhaus Berlin, ukb) is situated in the eastern periphery of the city, less than a 5-km distance from the city border. Inaugurated in 1997, the level I trauma center possesses a joined Institute of Radiology and Neuroradiology and provides care for more than 600 inpatient patients. More than 17 years ago, the administration and Department of Radiology initiated efforts to provide medical care beyond state borders and to define the hospital as a central partner for patient transfers. The idea of the ukb teleradiology network was born, and the first hospital was connected in 2004. During the following years, a team of confident radiologists and in-house and off-site technicians strove to enlarge, improve, and perfect the network continuously. At the end of 2020, the network included 23 small- to medium-sized hospitals of public and private bodies in three neighboring states (Brandenburg, Saxony, and Saxony-Anhalt; see Supplementary Table S1 for more details) with differing depths of technical integration and varying extensions of coverage. One large accident insurance consultancy and one mobile stroke unit with computed tomography (CT) became part of the network as well.

### 1.4. Intention

Recent literature provides an abundance of information on technical details, local and international laws and requirements for setting up and running a teleradiology service. However, during the growth process of a teleradiology network, many discouraging and heartening phases can be experienced, which shall be the main focus of the following project report.

In detail, we aim to summarize the growth of an inhomogeneous hospital-based teleradiology network. We identify obstacles and illustrate solutions driving the development of a national teleradiology network. Hereby, we intend to highlight innovations driven by teleradiology, the share of teleradiology in modern medical care, and their potential in linking medical experts and specialties despite geographical separation.

## 2. Evaluation

Some contents of this project report stemmed from objective sources derived from contracts, billing, monitoring, and controlling. However, the backbone of our research are the personal experiences of deeply involved staff members during 17 years of teleradiology at ukb. Qualitative and quantitative analyses were conducted in two steps. This report did not involve human subjects. Qualitative analysis was conducted solely among the coauthors of this report; thus, it was exempt from IRB review.

### 2.1. Qualitative Evaluation

#### 2.1.1. Semi-Structured Interview

In the first step, a junior staff member who was uninvolved in the implementation and extension of the teleradiology network recapitulated the positions and expertise of the senior staff members. Then, a unique interview guide was designed to ensure the equality of the respective fields and contributions to the teleradiology network. The format was based on the style of a semi-structured interview [24]. Three baseline questions were included to identify the position of the interview partner and her or his reference to the teleradiology network over time. The remaining five open-ended questions were meant to encourage personal accounts. The interviews were performed face-to-face in 30–60-min sessions. Four senior staff members, the coauthors of this report, participated in the survey. The interview guide contained the following questions:

(1) What is your position at ukb?
(2) How long have you been employed at the Institute of Radiology and Neuroradiology?
(3) When did you first encounter “teleradiology”?
(4) What were the three-five most important milestones during the development of this teleradiology network?
(5) Which are obstacles/barriers that you and the team encountered?
(6) What helped to overcome each of these obstacles—which solutions failed, and which solutions succeeded?
(7) Which technical innovations have facilitated the work processes?
(8) Which future innovations do you anticipate in the field of teleradiology?

The individual answers of the staff members were analyzed and summarized to elaborate on obstacles, facilitators, and solutions during the implementation and growth of the teleradiology network. Available and suitable literature was compared and discussed.

#### 2.1.2. Review of the Implementation Timeline and Technical Steps

The sequence of implementation and the year each hospital was connected to the network were retrieved from teleradiology contracts. Major technical innovations facilitating the integration of new sites and daily work routines were retrieved from the staff members’ answers to questions 4–7 of the interview and illustrated in a graphic timeline. The depth of integration (connection to ukb RIS, PACS, both or neither) at the end of 2020 was the major determinator for grouping the teleradiology sites. The extension of coverage (full-time or on-call duty) was also reported.

### 2.2. Quantitative Evaluation

Examination numbers were monitored routinely on a yearly basis at the study site. The results of the last ten years were examined by the main author, separated by imaging modality, and reported for in-house versus teleradiology examinations. PACS archiving numbers at the main site were obtained from the PACS provider. Additionally, the controlling department was contacted to review the number of patients treated at the study site annually. The rate of transferred patients was confined as well. Accounting and controlling units were asked to provide the annual profits generated through the teleradiology network. The results were confidentially expressed as relative values. Profits are represented by the gross hospital intake for all radiology reports in the teleradiology network.

Patient numbers, examination numbers, and profits were recorded in Excel sheets (Microsoft Office 2019, Microsoft Corporation, Redmond, WA, USA). Corresponding trend curves representing annual data were superimposed to provide a graphic correlation of the results in relative values.

## 3. Identification of Qualitative and Quantitative Factors for Success

### 3.1. Qualitative Factors

#### 3.1.1. Interview Results

The individual answers of the senior staff during the semi-structured interviews can be extracted from Supplementary Table S2. Elaborate results and summaries are contained in Table 1.

#### 3.1.2. Teleradiology Sites

**Figure 1:**
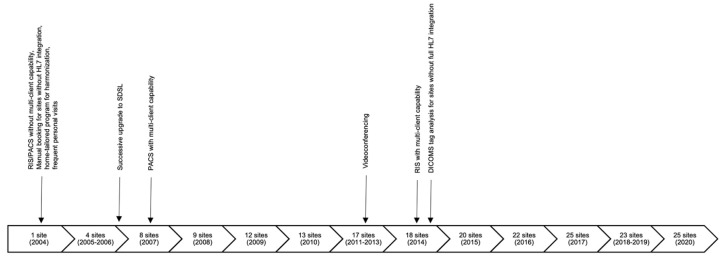
Timeline of integration of teleradiology sites and technical innovations.

In 2004, the first site was connected to the teleradiology network. At that time, RIS and PACS were robust systems but did not guarantee data protection through multiclient compatibility. Examinations had to be booked manually for sites without HL7 integration, and a home-tailored program was developed to harmonize the workflow for sites with an individual RIS. Thus, at that time, the teleradiology network was very inhomogeneous concerning technical integration and practical/clinical workflows. Additionally, frequent personal visits were necessary to perform clinical conferences and quality assurance and to educate clinicians and technicians. In the following years, symmetric digital subscriber lines (SDSLs) replaced integrated services digital networks (ISDNs) successively and radio-relay systems were expanded, which significantly reduced transmission times. A PACS with multiclient capability was purchased in 2007, and a modern RIS architecture with multiclient capability was introduced in 2014 after 15 sites were connected to the network. Staff requirements increased rapidly and were intensified due to personal visits at all individual sites up to twice per week. With the introduction of videoconferencing after eight years, frequent contacts could be continued while reducing personal visits to once per month at sites without radiologists. In 2014, a server for analysis of DICOM tags was set up to transfer relevant information to the main site’s RIS via HL7 ORM (order entry message) and thus render booking of examinations manually unnecessary for sites without full HL7 integration (Figure 1). From 2004 to 2020, a total of 30 sites and a mobile CT unit were connected to the network, and contracts with five sites were discontinued. Additionally, collaborations with further hospitals exist to exchange DICOM data for counseling purposes between radiologists and/or clinicians.

#### 3.1.3. Grouping of Teleradiology Sites

The depth of integration and the extension of coverage of all 25 sites at the end of 2020 were differentiated into three groups (Table 2). For sites without HL7 integration, only DICOM images are provided, and the radiology report has to be faxed. An export server is used to export DICOM tags and create an HL7 ORM. Data can then be transferred to the main site’s RIS and used to generate the radiology report. Four sites and one mobile CT scanner are currently connected to the network without HIL7 integration. However, full-time coverage is arranged only for the mobile scanner. Six sites with an individual RIS and HIS with HL7 integration are connected to the teleradiology network, 50% of which lack a local radiologist and are covered full-time. The introduction of modern RIS/PACS architecture enabled technical harmonization of the workflows. Before, a home-tailored program was used to increase feasibility for the staff. The radiology report still has to be transferred manually to the local RIS. Fourteen sites are deeply integrated into the network since they are connected using the main site’s RIS and only an individual HIS. Thus, manual transfer of the radiology report into the local RIS can be omitted.

### 3.2. Quantitative Factors

**Figure 2:**
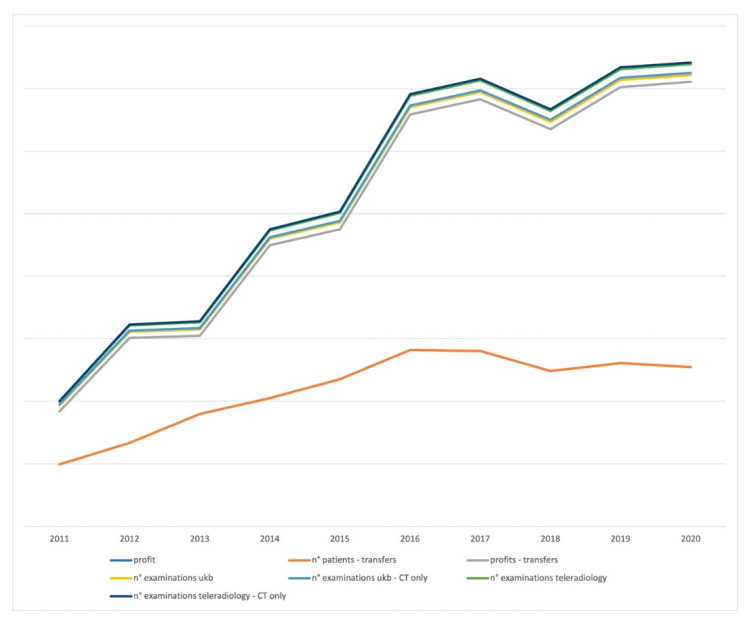
Graphic correlation of patients transferred from the teleradiology network to ukb, examination numbers, and teleradiology profits between 2011 and 2020 (x-axis). Profits were analyzed in Euros, and the results are confidentially illustrated as relative numbers (y-axis).

Examination numbers, patient volume, the percentage of patient transfers to ukb, and profits between 2011 and 2020 were recorded at the Department of Radiology and the controlling unit of the hospital. The results are displayed as curves with relative values to visualize the dependency of the items and their progress over time (Figure 2). Examination numbers, profits, and patient transfers increased from 2011 to 2012 after expanding the network from 13 to 17 sites (Figure 1). While patient transfers continued to rise, examination numbers and profits stagnated between 2012 and 2013 until more sites were connected, leading to an abrupt increase in examinations and profits until 2016 and 22 sites. The addition of three more sites in 2017 did not result in another leap in examination numbers, and patient transfers started to stagnate. In 2018, the number of sites was reduced to 23, leading to an incursion of examinations, profits, and patient transfers until they recovered in 2020 with the connection of two sites, for a total of 25 connected teleradiology sites. Parallel curve progression reveals that radiological imaging and patient volume are tightly linked because hardly any patient leaves a hospital without some sort of imaging. It must be discussed whether the increasing number of patients transferred to the main site might be coherent with the expansion of the teleradiology network, growing professional contacts in Berlin’s surrounding states, and a good reputation of the institute and the whole clinical team among the medical community.

## 4. Discussion

The manuscript on hand reports on the development of a hospital-based teleradiology network. It concentrates on describing obstacles and illustrates facilitators and solutions that enabled the success and growth of the project.

### 4.1. Technological Considerations

Technical developments in radiology paved the way for the success of teleradiology, and further technical innovations have been influenced significantly by the need for the improvement of teleradiology networks. Digitalization was one of the early obstacles the authors encountered. Initial costs slowed down the integration of the first site, so it was decided to allocate a few computed radiography systems from the main site. This decision facilitated the process of initiating the network. Cost-effectiveness and increasing profit, as shown for the teleradiology network at ukb, have been demonstrated frequently [25,26] and might justify the support of the smaller institution, as described for an international teleradiology project [19]. Examination numbers at sites providing teleradiology services naturally increase through adaptation and enlargement of a teleradiology network. The connection of institutions through teleradiology also promotes communication and cooperation between clinicians at different sites and bears the potential for growing patient transfers to the providers’ sites. Imaging can be reviewed, and patient transfers can be discussed in an interdisciplinary way during teleconferencing to avoid unnecessary transfers. However, digitalization and the maintenance of a teleradiology site are costly and can only be mastered through initial investments [27,28]. As an upside, CT and MRI have always been digital techniques [29]. CT imaging is increasingly being performed with ever-new indications of ultralow dose protocols competing with conventional radiography [30].

Image transmission times are relevant for efficiency, turn-around, and profits. In teleradiology networks involved in acute care, fast transmission of images of critically ill or injured patients is momentous. Image transmission issues decreased over time due to the spreading availability of faster data lines and standardized image compression [31]. Luckily, desperate considerations to reduce transmission time by sending only several selected images of a CT examination could eventually be dismissed [32]. Nevertheless, the spiral CT technique, generating more than a thousand thin slices during a whole-body scan, still poses difficulties on some data connections. Ultimately, investments in national infrastructure are key to the continued functionality of high-quality image transmission.

The complexity and heterogeneity of a teleradiology network can be overwhelming. Even teleradiology specialists need to be skilled in mastering interruptions of daily routine because of the necessity for time-consuming technical procedures during image evaluation and reporting. Self-studies, continuing education of the staff, and a background in computer science have empowered the ukb team to develop and maintain their intricate network. A home-tailored program, for instance, helped to overcome gaps in the integration of sites and harmonized the daily workflow. Developments of modern RIS/PACS structures later facilitated maintaining an overview of the system and further simplifying workflows after a difficult period of migrating systems [33].

The specific experience of in-house IT experts at the providers’ site and of off-site IT experts at the connected sites with teleradiology is vital. Close cooperation between radiology and IT departments is crucial as well. However, this cooperation might be endangered in the future by economic pressure and incorporation of hospitals with centralized IT departments and impersonal IT support [34].

### 4.2. Administrative and Practical Considerations

Each signed teleradiology contract is preceded by negotiations between managers. Personal dialogue can reduce misunderstandings and was essential during the early stages of network development when teleradiology was new to hospital managers in Berlin’s surrounding areas. After connecting the first few sites to the network and setting precedents, gaining a reputation, and word-of-mouth recommendations, persuasion on the management level became easier, and the teleradiology network grew continuously from 2004–2020 in the eastern part of Germany. Expansion to the western areas was limited due to pronounced competition by teleradiology providers based on outpatient care. However, geographic proximity to the providers’ site has proven to be essential to surpass a mere reporting function and to link clinical subspecialties of hospitals. In 2018, contracts with two hospitals at the western border of Berlin were discontinued after seven years of teleradiology service to enable new cooperation. It had to be acknowledged that the connection with these hospitals had not developed into profound cooperation on the clinical level. Both sites were located closer to another tertiary trauma center to which patients were transferred if necessary.

Regulative requirements and data protection aspects can be other major obstacles for teleradiology. It must be considered that those demands have been implemented to ensure patient safety. Nevertheless, officials were inexperienced in the field of teleradiology in the beginning and thus susceptible to questionable demands. Legal aspects and data protection requirements busied teleradiology communities worldwide and promoted the publication of various articles [35,36,37,38,39,40,41,42,43,44,45]. The senior authors described that close collaboration with officials and involvement in the development of regulations from the beginning of teleradiology networking in Berlin significantly eased cooperation in the years to come. The employment of a medical physics expert (MPE) in 1997, long before it became mandatory in 2019 in Germany, and the implementation of dose-management software further facilitated processes with low friction. Data protection was challenging initially, with underdeveloped RIS/PACS architectures, but improved with multiclient capability. However, some sites are still connected without HL7 integration, which is debatable from a data protection point of view since it demands faxing radiology reports. Fully integrated sites, as well as innovative and secure communication platforms, should solve this issue in the future.

Harmonization of workflows was one of the most pressing issues for senior staff members concerning teleradiology. The inhomogeneity of the network concerning the depth of integration and coverage resulted in setups with independent local radiologists and sites with unsupervised technicians. These circumstances became increasingly challenging with the growth of the network. Such obstacles have already been identified by other authors, but facilitators and solutions should be named here anyhow [14,46]. The modernization of the RIS and the establishment of videoconferencing were technical approaches to the needs of daily routine. Nonetheless, the flexibility of the staff at the main site and connected hospitals to perform and participate in recurrent training were key factors in the harmonization process. Close interpersonal relationships with frequent visits of a dedicated contact person earned the trust of technicians and clinicians to align to given workflows in a stepwise fashion. Future technical innovations in automated case management and process monitoring might have the potential to further simplify oversight in teleradiology networks.

Videoconferencing was also relevant in stabilizing staff requirements despite the growing number of connected hospitals and has been described before implementation at ukb in 2012 [47]. Teachings and conferences via videoconferences could reduce the number of personal visits at teleradiology sites. The high frequency of consultations by phone, one of the major services of the teleradiology network on-hand, and beneficial access for clinicians and technicians to the staff, are still time-consuming and uncompensated by insurance. This issue still needs to be addressed in discussions about reimbursement and should be brought to the attention of developers of modern communication platforms. Until then, the workload, which is increased by rising examination numbers and fewer tangible factors, has to be faced by growing teams [48]. AI solutions can support staff members in prioritizing work, and highly functional home office setups could deskew personnel to enable continuous teleradiology coverage even in times of crisis, such as the recent pandemic.

## 5. Conclusions

### The Importance of Hard and Soft Facilitators

The current project report mentions various hard and soft facilitators in the development of a national hospital-based teleradiology network. In the revision of each topic, we learned that obstacles were often solved by hard factors or technical innovations. Many times, the time span until the development of such a solution required personal efforts of the teleradiology staff, endurance, and flexibility of local and external team members. Some issues remain unsolved, and some obstacles have yet to be overcome. Despite the technicality of teleradiology per se, personal relations, the ability to connect all medical specialties through specialized consultancy, and close interpersonal collaborations were keys to the success of this project and will remain a unique feature and selling point of this teleradiology network.

## Figures and Tables

**Table 1:** Obstacles, facilitators, and solutions during network implementation and expansion.

Topic	Goal	Obstacle	Soft/Hard Facilitator(s)	Solution	Future Goals/Unsolved Issues
Imaging format	Digitalization	- Initial costs of equipment - Changed workflow requirements	- Allocation of computed radiography systems to first site ^h^	- Investments	n/a
Data transmission	Acceleration	- Adequate transmission times in-house but insufficient inter-institutional	- Upgrade of local and national data streams ^h^ - Stepwise transmission of images ^h^	- Radio relay system/SDSL ^ps^ - Compressed image transmission ^su^	- Continuous upgrade of national infrastructure
Technical complexity	Overview, simplification	- Variability of technical systems at sites (RIS/HIS/PACS)	- Learning curve of staff at main site ^s^ - Close cooperation of radiology department and local IT specialists ^s^	- Specific talents/skills/education of staff concerning technical aspects ^su^	n/a
Management negotiations	Persuasion, productive collaboration, expansion of the network, adequate reimbursement/profit	- Incomprehension/inexperienced management at sites	- Continuous personal contacts/dialogue ^s^ - Increasing personal experience of senior staff ^s^	- Precedent-setting ^su^ - Reputation/propaganda ^su^	n/a
Regulative requirements/restrictions (laws)	Safety versus feasibility	- Inexperience of authorities	- Participation/involvement in the development of regulations from the beginning ^s^	- Employment of a Medical physics expert (MPE) - dose-management software	
Data protection	Safety	- Initially underdeveloped RIS/PACS architecture - Low integration depth at some sites➔faxing of reports	- Fax servers with programmed sites ^h^	- Multi-client capability of RIS/PACS ^su^ - HL7 integration of most sites ^ps^	- Reduction of telephone calls/fax reports through innovative communication platforms
Workflows	Harmonization	- Missing HL7 integration at some sites - Local independent radiologists - Physical distance to technicians and clinicians	- Home-tailored program with HL7 ORM ^h^ - Personal contact/communication with local staff ^s^	- Modern RIS architecture at main site ^su^ - Limitations in case of differing external RIS ^ps^ - Videoconferencing combined with personal visits depending on a site’s needs (flexibility) ^ps^ - Recurrent training of local staff ^su^ - Dedicated contact person for external staff ^su^ - Stepwise alignment of workflows ^su^ - Acquisition of all radiologic duties/ radiologic “serenity” at some sites ^ps^	- Standardization/harmonization of differing RIS solutions - Technical solutions specifically tailored for radiologists’ needs - Whole process offer from counseling/indication to radiology report and recommendations to increase patient transfers to the main site - Monitoring of network by AI: technical system analysis and management of cases
Network size	Expansion	- Competition	- Personal support and long-lasting experience as unique selling points compared to newer competitors ^s^ - Broad clinical experience of radiologists compared to competitors based in outpatient care ^s^	- Focus on eastern parts of the country with less coverage ^ps^ - Reputation/propaganda ^su^	n/a
Workload	Patient safety, prioritization, anticipation of exceptional circumstances (i.e., pandemic)	- Increasing examination numbers - Fewer radiographs and more complex CT imaging - Reduced home office functionality	- Precise briefings between radiologists and external clinicians (via phone) and in-between shifts ^s^	- Increased workforce, double occupancy during on-call hours ^ps^ - Artificial intelligence, algorithms to support radiologists ^ps^	- Improvement of AI - Innovative communication platforms - Adaptation of salary laws to enable billing of multilateral communication - Complete functionality in home offices to enable rectification, growth
Staff requirements	Stability	- Frequent personal visits - Frequent consultations by phone	- Videoconferencing ^h^	- Videoconferencing combined with personal visits depending on a site’s needs (flexibility) ^ps^	- Improvement of AI - Innovative communication platforms - Adaptation of salary law to enable billing of multilateral communication

^s^ soft facilitator, ^h^ hard facilitator, ^ps^ partial success, ^su^ success.

**Table 2:** Depth of integration (Grouping of sites into depth of integration and coverage by main site).

	Sites without HL7 Integration	Sites with Individual RIS and HIS	Sites with Connection to Ukb RIS and Individual HIS
Number of sites	4 (+mobile CT)	6	14
Full-time coverage	1 (mobile CT)	3	8
Current relevance	➔ Creation of an export server that extracts patient data from DICOM tags and creates an HL7 ORM to transfer the data to the RIS. The radiology report has to be faxed.	➔ The introduction of a modern PACS and RIS harmonized the workflow before the use of a home-tailored program was required. The radiology report has to be transferred into the local RIS manually.	➔ No manual transfer of radiology reports into RIS necessary at individual sites.

## Data Availability

Data are contained within the article or supplementary material.

## Supplementary Materials

The following are available online at https://www.mdpi.com/article/10.3390/healthcare9121684/s1. Supplementary Table S1. Summary of teleradiology hospitals in 2020; Supplementary Table S2. Individual answers during semi-structured interviews.
