# Supplementary material for: Obstacles and Solutions Driving the Development of a National Teleradiology Network
Source: Healthcare (Basel). 2021 Dec 6;9(12):1684. doi: 10.3390/healthcare9121684 (PMC8701208; doi:10.3390/healthcare9121684)
Supplement: Supplementary file 1 [file healthcare-09-01684-s001.zip › healthcare-1474121-supplementary.pdf]

Suppl. Table S1. Summary of teleradiology hospitals in 2020.

Site	Size (number of beds)	Level of care
#1,2	387	basic /intermediate
#3	171	basic
#4,5	270	basic
#6	170	basic
#7	165	basic
#8	55	basic
#9	140	basic
#10	250	intermediate
#11	100	basic
#12	245	intermediate
#13	90	basic
#14	96	basic
#15	306	intermediate/teaching hospital
#16	132	basic
#17	134	basic
#18	186	basic
#19	267	intermediate
#20	60	basic
#21	263	intermediate
#22	30	basic
#23	661	teaching hospital
Total	4.178	

Suppl. Table S2. Semi-Structured interview questions and individual responses of four senior staff members.

Question	Individual 1	Individual 2	Individual 3	Individual 4
#1	director	Deputy director (radiologist and computer scientist)	Senior physician, head of staff unit "Teleradiology" (radiologist)	x-ray technician, member of staff unit "Teleradiology"
#2	1997	1997	1997	2004
#3	2003 after affiliation to smaller hospitals without sufficient coverage by radiologists	2004 when the first hospital was to be connected	2004 when the first hospital was to be connected	2005 as x-ray technician with bookings
#4	(1) Digitalization of imaging<br>(2) Increasing network capacity<br>(3) Increasing standardization (DICOM, HL7)<br>(4) Increasing knowledge of regulating authorities, laws	(1) Connection of the first hospital<br>(2) Connection of the first hospital with full integration of RIS/PACS, prior digitalization<br>(3) development of first regulatory standards in Teleradiology with authorities<br>(4) switch from weekly on-site conferences to videoconferencing→less staff requirements	(1) Connection of the first hospital without external support<br>(2) Faster transmission rates (SDSL)<br>(3) Videoconferencing	(1) Digitalization of imaging<br>(2) Implementation of RIS with multi-client capability<br>(3) Videoconferencing
#5	- slow data transmission<br>- incomprehension of management boars of potential sites<br>- price negotiations<br>- competition through radiology practices involved in in- and outpatient patient care	- slow data transmission<br>- missing multi-client capability<br>- differing workflows for hospitals with individual RIS	- slow data transmission<br>- local radiologists with different workflows<br>- need for continuous guidance of technicians and clinicians (especially at sites without local radiologist)	- great variability of technical systems at sites<br>- differing workflows<br>- complexity of teleradiology network<br>- initial costs
#6	- radio-relay systems (partial success, in some areas still imperfect transmission)<br>- personal contacts/dialogue (success)<br>- increasing personal experience (success)<br>- employment of a Medical physics Expert and dose-management software (success)<br>- precedent-setting (success)<br>- focus on eastern parts of Germany→less (partial success, two sites were lost)	- switch from ISDN to SDSL for quicker transmission (partial success, in some areas still imperfect transmission)<br>- next generations of PACS and RIS (success)<br>- home-tailored program with HL7 ORM to harmonize workflow between different RIS (success), then next generation RIS	- switch from ISDN to SDSL for quicker transmission (partial success, in some areas still imperfect transmission)<br>- recurrent personal visits and exchange with local radiologists / complete acquisition of radiologies for continuous routine and emergency coverage (success)<br>- recurrent trainings of staff and conferences at sites during personal visits especially in absence of local radiologists	- specific talents/skills/education of staff concerning technical aspects (success)<br>- dedicated contact person for external staff→stepwise alignment of workflows (success)<br>- close cooperation of radiology department and local IT specialists (success)<br>- sale of digital x-ray equipment from ukb to teleradiology sites (success)
#7	- PACS and RIS architecture →multi-client capability, guaranteed data protection	- compressed image transmission (wavelet)<br>- SDSL<br>- PACS and RIS with multi-client capability	- DICOM and HL7 standards<br>- SDSL<br>- digitalization<br>- PACS and RIS with multi-client capability	- RIS with multi-client capability →full integration with data protection<br>- videoconferencing → omission of high-frequency personal visits

#8	- support/ prioritization by artificial intelligence<br>- innovative communication platforms to replace numerous phone calls<br>- adaptation of salary law to account for time-consuming multilateral communication	- complete acquisition of most sites from counseling/indication to radiology report and recommendations in case of transfers<br>- complete functionality in home offices→rectification, growth, anticipation of situations like pandemic	- systems focusing on radiologists<br>- artificial intelligence for problem analysis, management of cases	- Monitoring of the network possibly based on artificial intelligence to defect malfunctions
